# Quadruple Valve Replacement for Carcinoid Heart Disease

**DOI:** 10.21470/1678-9741-2017-0224

**Published:** 2018

**Authors:** Syed Saleem Mujtaba, Stephen Clark

**Affiliations:** 1 Freeman Hospital Newcastle, United Kingdom of Great Britain and Northern Ireland.

**Keywords:** Carcinoid Heart Disease, Malignant Carcinoid Syndrome, Heart Valves/surgery, Heart Valve Prosthesis Implantation

## Abstract

**Introduction:**

Carcinoid heart disease most frequently involves the tricuspid or, more rarely, the pulmonary valve and presents with right heart failure as 5-HT is metabolized by the lung. Left-sided valve involvement is quite rare. We describe our experience of 3 patients presenting with heart failure secondary to carcinoid heart disease affecting all four cardiac valves. There are only four previous isolated case reports in the literature.

**Methods:**

All three patients underwent quadruple valve replacement during a single operation. Right ventricular outflow tract reconstruction with a pericardial patch was performed in all patients. For 24 hours prior to surgery, all patients received intravenous octreotide, which continued in intensive care for at least 24 hours.

**Results:**

Mean cross-clamp and bypass times were 175 (range 164-197 minutes) and 210 (range 195-229 minutes) minutes, respectively. Mean intensive treatment unit (ITU) and inpatient stays were 2.3 (range 2-3 days) and 12 (range 9-16 days) days, respectively. One patient was reopened for bleeding 4 hours postoperatively from a ventricular pacing wire site. None required a permanent pacemaker postoperatively. There were no other complications in any patient. The quality of life was excellent at 6-16 months clinic follow-up as they were in NYHA 1. Postoperative echocardiography showed no paravalvular leaks and well-functioning prostheses in all cases.

**Conclusion:**

Surgery to replace all four valves is feasible with excellent medium-term survival and a very low rate of complications. Patients with carcinoid heart disease should always be considered for surgery irrespective of the extent of valvular involvement.

**Table:** 

Abbreviations, acronyms & symbols	
CT	= Computed tomography
NYHA	= New York Heart Association
PFO	= Patent foramen ovale

## INTRODUCTION

Carcinoid heart disease is a rare cause of intrinsic right heart valve disorders, leading to right heart failure. Left-sided heart valves involvement is occasional. Cardiac involvement in carcinoid disease generally results in tricuspid insufficiency and pulmonary stenosis. Carcinoid tumours are neuroendocrine malignancy. Ninety per cent of all carcinoid tumours are located in the gastrointestinal system, of which the most common sites are the appendix and terminal ileum^[1]^. Carcinoid tumours must be invasive or metastasise to produce carcinoid syndrome, which is characterized by facial flushing, diarrhea, and bronchospasm. The incidence of carcinoid tumours is approximately 1 in 75,000 of the population^[2]^, of whom about 50% develop carcinoid syndrome. Once carcinoid syndrome has developed, approximately 50% of these patients will go on to develop carcinoid heart disease or Hedinger syndrome.

## METHODS

We describe our experience with 3 patients who underwent simultaneous quadruple valve replacement for carcinoid heart disease. Patients were in mid 50's (mean 55.3, range 55-56 years, male=1, female=2). All had been diagnosed with a small bowel carcinoid tumour with liver metastases having initially presented with abdominal pain, flushing and dyspnoea [New York Heart Association (NYHA) functional class 3-4]. Computed tomography (CT) of the abdomen revealed multiple liver metastases and a small bowel mesenteric mass. A laparoscopic biopsy was consistent with a low-grade carcinoid tumor. All had good medium-term estimated prognoses but were deteriorating rapidly due to congestive heart failure.

**Fig. 1:**
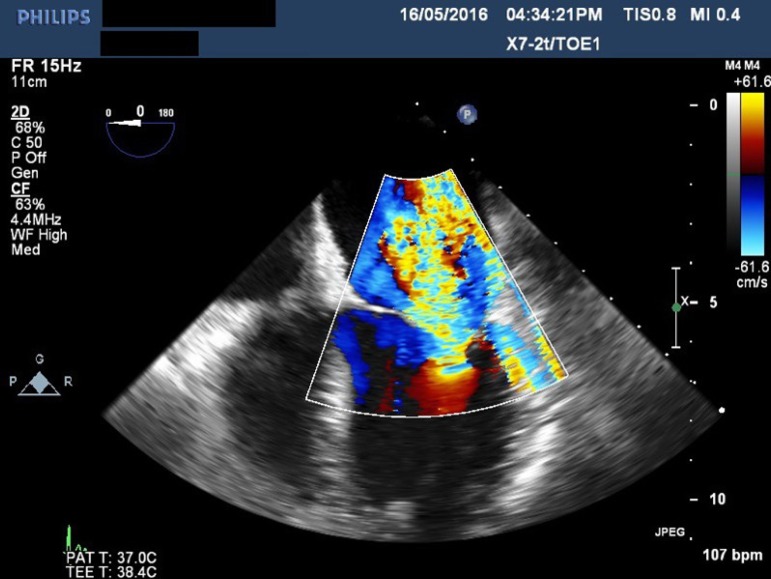
Transesophageal echocardiogram showing severe mitral regurgitation.

**Fig. 2:**
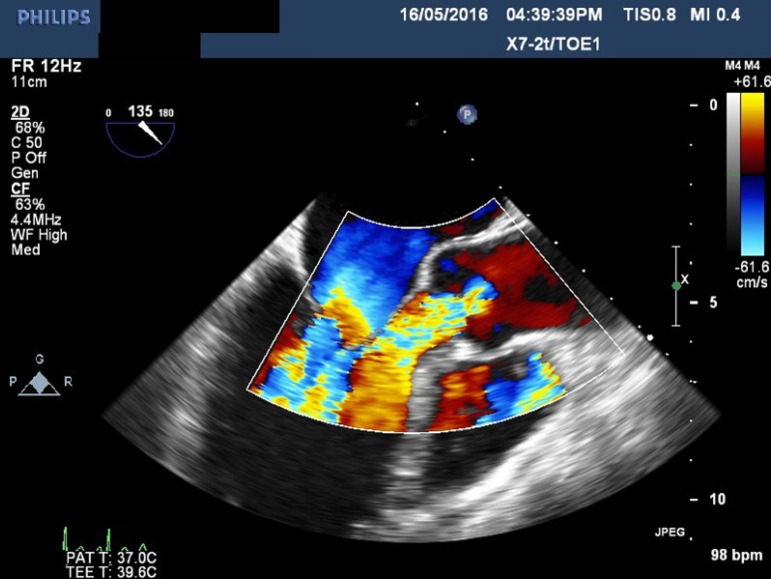
Transesophageal echocardiogram showing massive aortic regurgitation.

**Fig. 3:**
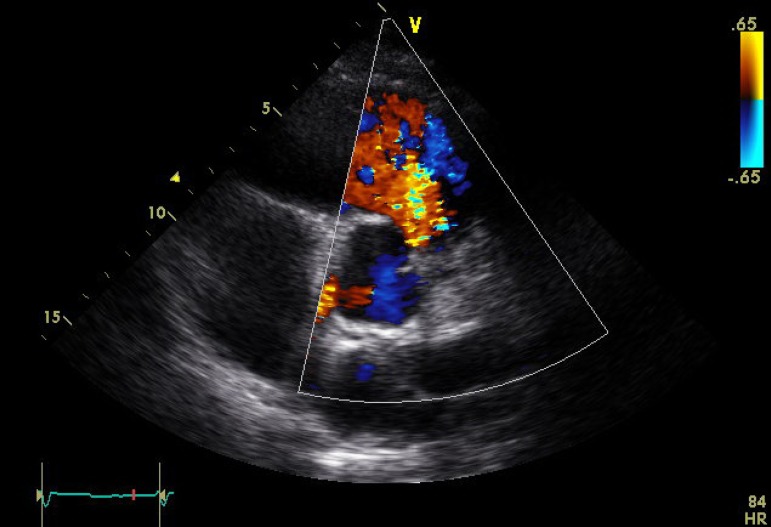
Transthoracic echocardiogram showing torrential pulmonary regurgitation.

**Fig. 4:**
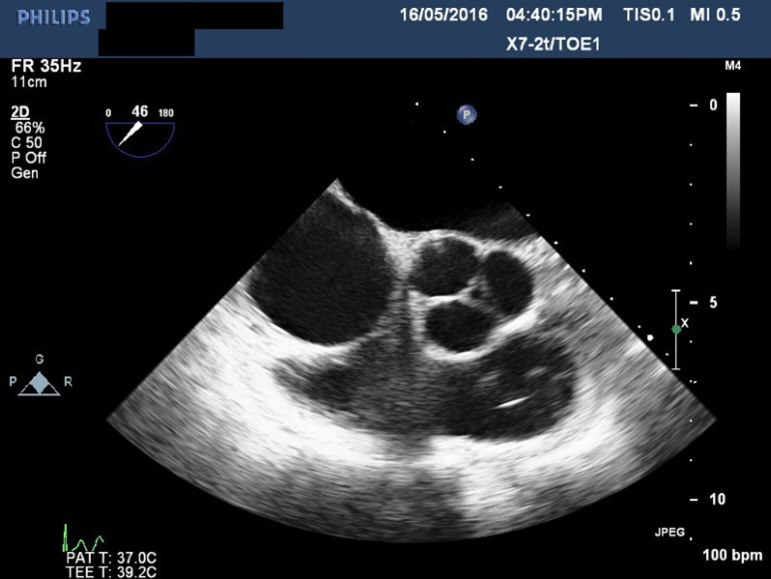
Transesophageal echocardiogram showing thickened aortic valve leaflets.

**Fig. 5:**
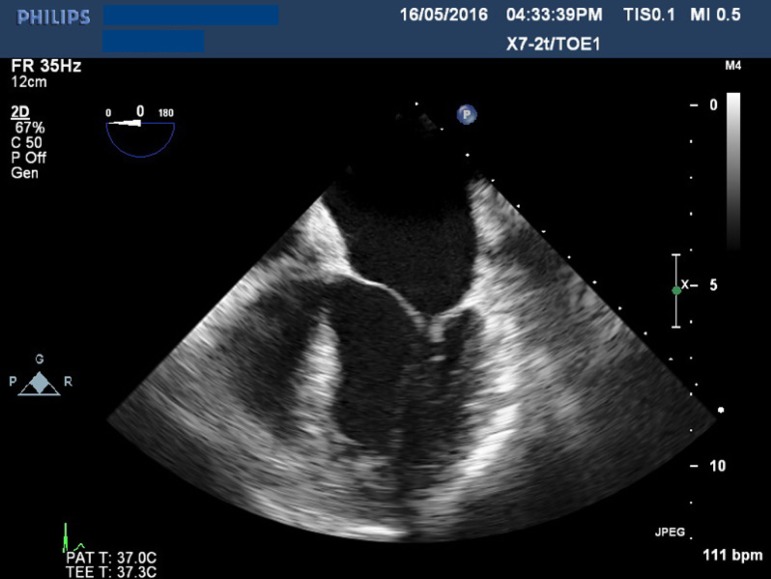
Transesophageal echocardiogram showing severely thickened mitral valve leaflets.

**Fig. 6:**
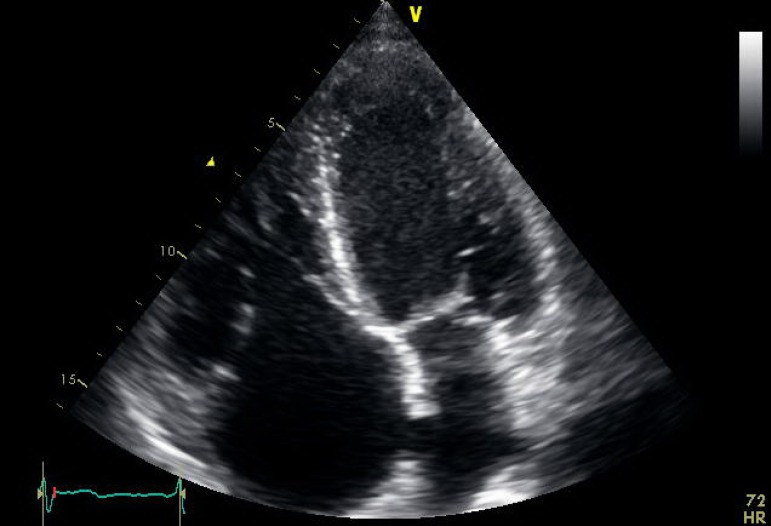
Transthoracic echocardiogram showing severely thickened tricuspid valve leaflets.

Examination of all patients revealed an elevated jugular venous pressure with prominent 'V' waves and bilateral pedal oedema. On auscultation, there were systolic murmurs over the apex and left fourth intercostal space. Early diastolic murmurs were present at the upper and lower left sternal edges. Echocardiography revealed advanced features of heart failure. All four cardiac valve leaflets were fixed, thickened, retracted and failed to coapt. There was at least moderate to severe regurgitation of all four valves (Figures 1 to 3), except in one patient who had mild tricuspid regurgitation but thickened fixed leaflets consistent with carcinoid involvement (Figures 4 to 6). A patent foramen ovale (PFO) was not found in any patient. The left ventricular function was preserved in two and moderate in the third patient (mean 50%, range 40-55%), while the right ventricle was dilated with preserved function. The coronary arteries were disease-free on angiography. Mean logistic EuroSCORE was 4.1 (range 2.04-7.41).

Because of the severity of cardiac symptoms, given that there estimated life expectancy was more than two years and based on the course of their carcinoid disease, the patients were accepted for surgery.

**Fig. 7:**
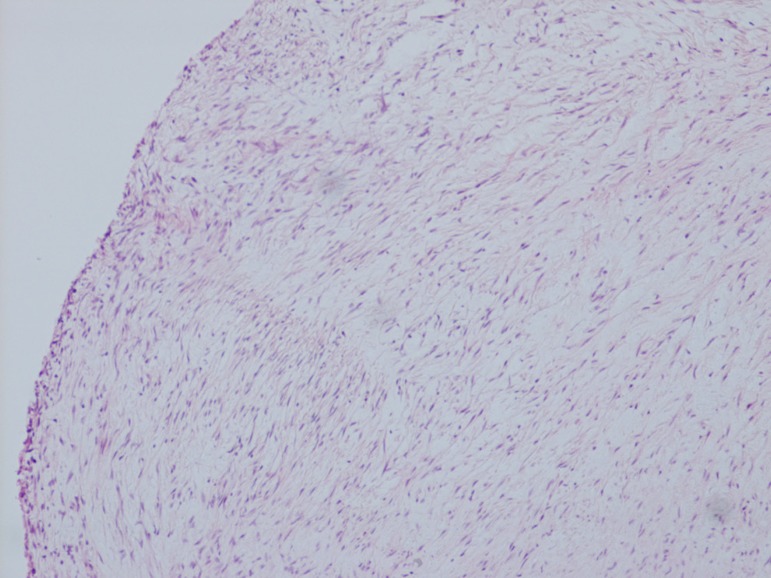
Histology slide of carcinoid aortic valve.

**Fig. 9:**
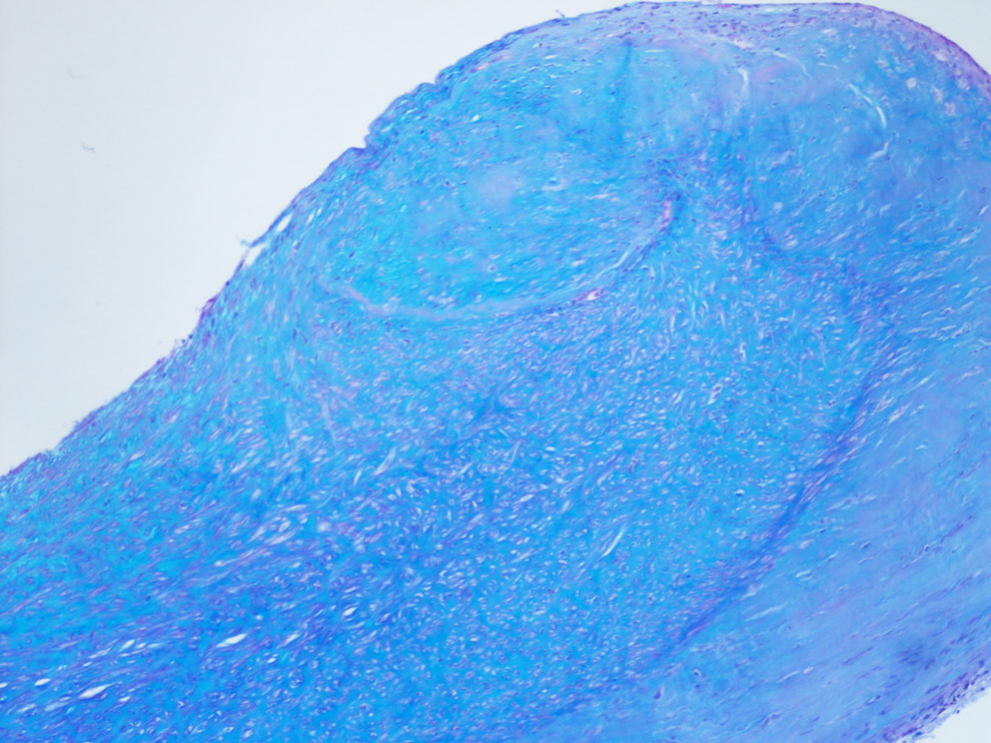
Histology slide of carcinoid pulmonary valve.

An intravenous infusion of octreotide (600 µg in 60 mL 0.9% saline) was commenced a day before surgery at a rate of 5 mL/h, delivering 50 µg/h octreotide. This continued during surgery and stopped 48 hours after the operation. Surgery was performed with the patient on aortobicaval cardiopulmonary bypass via a midline sternotomy. The patient's systemic temperature was lowered to 32ºC, with myocardial protection being achieved through antegrade cold blood cardioplegia, which was repeated every 30-45 minutes. All excised valves had a classical appearance characteristic of carcinoid heart disease (Figures 7 and 9).

**Fig. 10:**
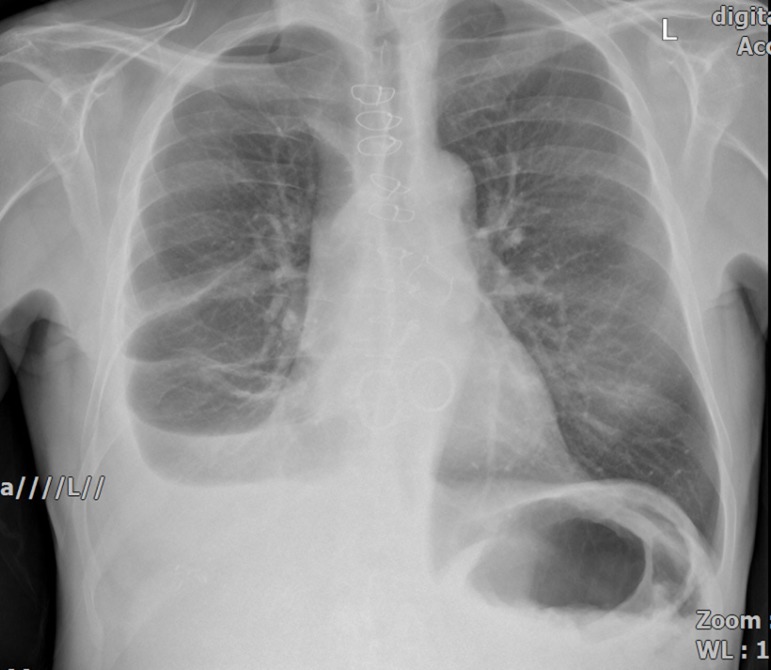
Chest X-ray showing all four replaced valves.

In two patients, the aortic and mitral valves were replaced with CarboMedics mechanical valves while the tricuspid and pulmonary valves were replaced with Perimount Magna bioprostheses (Figure 10). This permitted the use of a postoperative pulmonary artery flotation catheter and given the durability of bioprostheses in the right heart. In the third patient, all valves were replaced with Perimount Magna bioprostheses. Right ventricular outflow tract reconstruction (pulmonary augmentation) with a pericardial patch was performed in all patients.

## RESULTS

The mean cardiopulmonary bypass time was 211 min (range 195-229 min) and the cross-clamp time 179 min (range 164-197 min). Patients were transferred to the intensive care unit in a hemodynamically stable condition, with a modest dose of adrenaline and noradrenaline, and all were extubated on the night of their surgery. They were temporarily paced for four days with subsequent recovery of their intrinsic sinus rhythm and the pacing was discontinued. None required a permanent pacemaker. None had any other postoperative complications what so ever, except for one patient who required re-exploration 4 hours postoperatively for bleeding from a right ventricular temporary pacemaker site. All were discharged home in a stable condition.

The mean intensive care unit stay was 2 days while the mean hospital stay was 12 days (range 9-16) (Table 1). Postoperative echocardiography showed all prostheses were seated well with no paravalvular leaks. All excised valves had pathologic findings consistent with carcinoid heart disease. They were followed for 6-16 months (mean 12 months) in the outpatient clinic and all were NYHA class 1. One patient went on to ileal carcinoid tumour and hepatic metastasis resection.

**Table 1:** Outcomes of patients with carcinoid heart disease who underwent quadruple valve replacement.

Patient	1	2	3
Age (years)	56	55	55
Gender	Male	Female	Female
NYHA preoperative	3	4	3
NYHA postoperative	1	1	1
Surgery	August 2015	November 2015	August 2016
Follow-up (months)	16	14	6
PFO	No	No	No
X-clamp (minutes)	177	197	164
Bypass (minutes)	209	229	195
Aortic regurgitation	Moderate-severe	Moderate-severe	Severe
Valve implanted	23 mm CarboMedics	23 mm Perimount Magna	19 mm CarboMedics
Mitral regurgitation	Moderate-severe	Severe	Severe
Valve implanted	27 mm CarboMedics	25 mm Perimount Magna	27 mm CarboMedics
Tricuspid regurgitation	Severe	Severe	Mild
Valve implanted	29 mm Perimount Magna	25 mm Perimount Magna	25 mm Perimount Magna
Pulmonary regurgitation	Severe	Severe	Moderate-severe
Valve implanted	23 mm Perimount Magna	21 mm Perimount Magna	23 mm Perimount Magna
EuroSCORE	4	7	3
Logistic EuroSCORE	2.86	7.41	2.04
Left ventricle	Good (EF 55%)	Moderate (EF 40%)	Good (EF 55%)
Right ventricle	Ventricle dilated	Dilated	Ventricle dilated
ICU stay (days)	2	2	3
Hospital stay (days)	9	11	16
Chest drain (mL)	2,200	250	250

EF=ejection fraction; ICU=intensive care unit; NYHA=New York Heart Association; PFO = patent foramen ovale; X-clamp=aortic cross-clamp

## DISCUSSION

The cardiac manifestations are caused by 5-hydroxytryptamine (5-HT or serotonin) released by the malignant cells rather than any direct metastatic involvement of the heart. Preferential right heart involvement is most likely related to inactivation of the vasoactive substances by the lungs. In 5-10% of cases with left-sided valvar pathology, one should suspect extensive liver metastases, bronchial carcinoid, or a patent foramen ovale^[3,4]^.

Circulating serotonin levels are more than twofold higher and urine levels of the serotonin metabolite 5-HIAA are almost fourfold higher in the carcinoid heart disease patients compared with the noncardiac group. Elevated serum serotonin is 100% sensitive, but only 46% specific for carcinoid heart disease^[5]^.

Patients with carcinoid syndrome should be treated in specialized centers by a multidisciplinary team, including oncologists, cardiologists, pathologists, and surgeons^[6]^. Without intervention, patients may develop progressively worsening symptomatic right heart failure. Life expectancy is significantly reduced as a result when prognosis otherwise from the carcinoid tumour may be good in the medium- to long-term. The Mayo Clinic showed a mean life expectancy of 1.6 years for those with the cardiac disease compared with 4.6 years for those without cardiac disease in patients with metastatic midgut carcinoid tumours^[7]^.

Medical management is directed by reduction of symptoms caused by right-sided heart failure and includes fluid and electrolyte management, diuresis, and enhancing cardiac function. This can be achieved with the use of digoxin, loop and thiazide diuretics, salt and fluid restrictions, and continuation of somatostatin analogues^[4]^. Treatment with octreotide gives rise to both directly observable clinical benefit and measurable biochemical improvement. About 70% of patients obtain symptomatic relief from diarrhea and flushing, showing a decrease in measurable 5-HIAA urinary secretion and serum 5-HT concentrations^[8]^. There is some evidence that the use of leucocyte interferon-alpha controls the secretion of tumour products, can produce a notable reduction in tumour size with evidence of survival benefit. Unfortunately, however, there are no data to suggest that either interferon or octreotide can cause any regression of the cardiac damage caused by carcinoid disease. Cytotoxic chemotherapy is given for extensive disease and dual endothelin receptor antagonist like bosentan prevents valvular and mural fibrosis and improve heart function^[9]^.

Cardiac surgery offers marked symptomatic improvement of >1 New York Heart Association class after valve replacement^[10,11]^. There also may be survival benefit with cardiac surgery, although this is difficult to prove, given the other morbidities in this patient group. The surgical indications of carcinoid heart disease are symptomatic right ventricular failure, severe valvular dysfunction and systemic venous pressure elevation^[12]^. Surgery is contraindicated in end-stage metastatic disease, patients with poorly controlled carcinoid symptoms despite octreotide therapy, or hepatic dearterialization^[13]^. Median survival after cardiac valve replacement varies between 6 and 11 years^[14]^. The improved survival of patients with congestive cardiac failure in recent years may reflect the increasing surgical expertise in this field and better perioperative management of the patient with octreotide^[6]^.

More controversial is the choice of the valve prosthesis. Problems for homograft use for pulmonary valve replacement are homograft constriction, homograft calcification and homograft plaque deposition. In most cases, the involvement of valve leaflets is too extensive to allow surgery for commissurotomy or valvotomy, and pulmonary valvectomy surgery is required^[15]^. On the one hand, biological prosthetic valve degeneration with valve allograft failure has been reported to occur as early as 3 months after implantation, being the result of intractable high levels of NET vasoactive products inducing carcinoid plaque reformation^[16-18]^. Evidence for this, however, is relatively weak. On the other hand, the use of mechanical prosthesis requires life-time anticoagulation in these patients who have an already increased risk of bleeding due to hepatic dysfunction. In addition, the risk of mechanical tricuspid mechanical prosthesis thrombosis is approximately 4% per year^[19]^. Therefore, choice of the prosthesis should be tailored to the individual patient risk of bleeding, life expectancy, and future interventions. An additional consideration is the ability to insert a pulmonary artery flotation catheter postoperatively through a biological prosthesis to assist management especially after multivalve replacements in patients with pre-existing cardiac failure. Although surgical valve replacement is the gold standard treatment for symptomatic carcinoid heart valve disease, transcatheter pulmonary valve replacement should be considered as an alternative approach in high-risk candidates^[20]^. Percutaneous valve implantation in the pulmonary and in the inferior vena cava positions may offer minimally invasive alternative in the high-risk patients^[21]^.

Mabvuure et al.^[22]^ cautiously concluded that biological valves have an acceptable lifespan in patients with carcinoid syndrome and that the process of valve destruction seen in carcinoid patients does not continue to a significant level in the bioprosthesis. Although there opinion lacks, any direct comparative trial or higher-level evidence is predominantly based on case reports.

The present article highlights three cases of simultaneous quadruple valve replacement in for carcinoid heart disease, and further supports the possibility of not only successful quadruple valve replacement, but also an excellent postoperative outcome for patients with a reasonable life expectancy and well-controlled disease. Complications are few and management is aided by perioperative octreotide infusion.

## CONCLUSION

Quadruple valve replacement is an acceptable option for patients with a good prognosis and symptomatic carcinoid heart disease affecting all four heart valves. An experienced medical, surgical, and anesthetic team approach to the patient with carcinoid heart disease is critical to provide state-of-the-art management for these patients. Excellent outcomes can be achieved with low risk of complications.

**Table:** 

Authors' roles & responsibilities	
SSM	Substantial contributions to the conception or design of the work; or the acquisition, analysis, or interpretation of data for the work; final approval of the version to be published
SC	Substantial contributions to the conception or design of the work; or the acquisition, analysis, or interpretation of data for the work; final approval of the version to be published

## Figures and Tables

**Fig. 8:**
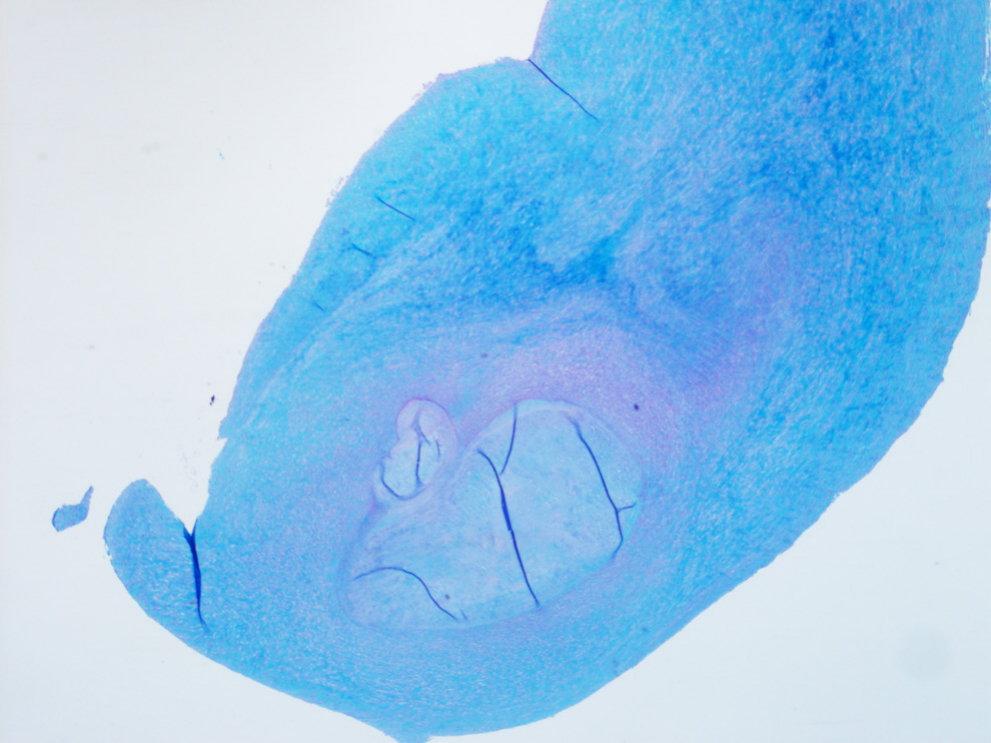
Histology slide of carcinoid mitral valve.
